# Comparing the Outcomes of Laparoscopic Versus Open Surgery for Colorectal Cancer: A Systematic Review and Meta-Analysis

**DOI:** 10.7759/cureus.90687

**Published:** 2025-08-21

**Authors:** Yazan Mahmoud, Varadheswari Thiagarajan, Yousif Jihad, Akash Ranganatha, Fatima Khalid, Binish Essani

**Affiliations:** 1 Surgery, University Hospitals of Morecambe Bay NHS Foundation Trust Cumbria, Kendal, GBR; 2 Internal Medicine, Government Virudhunagar Medical College Hospital, Virudhunagar, IND; 3 Orthopedic Surgery, United Lincolnshire Hospitals NHS Trust, Lincoln, GBR; 4 Internal Medicine, JJM Medical College, Davangere, Davangere, IND; 5 Internal Medicine, King Edward Medical University, Lahore, PAK; 6 Medicine, Jinnah Medical and Dental College, Karachi, PAK

**Keywords:** colorectal cancer, laparoscopic surgery, meta-analysis, open surgery, short-term recovery

## Abstract

Colorectal cancer (CC) is a leading source of cancer-related morbidity and mortality globally. Laparoscopic surgery (LP), a minimally invasive approach, has gained popularity as a substitute for open surgery (OP) due to its potential benefits in recovery. This systematic review and meta-analysis sought to assess the relative outcomes of LP as opposed to OP for CC. The main interest was in the short-term outcomes (STO) of recovery, including blood loss, hospital stay, and postoperative complication. A systematic search of various databases (Cochrane Library, PubMed, Web of Science, Google Scholar) was performed to include cohort studies and randomized controlled trials (RCTs) from 2010 to 2025. Pooled analysis was done using a random-effects model, and heterogeneity was determined using the I² statistic. Publication bias was tested with funnel plots and Egger's test. The meta-analysis displayed that LP had significantly reduced hospital stays, less intraoperative blood loss, and fewer postoperative complications than OP. The pooled effect size for short-term recovery outcomes showed a moderate significant positive effect of LP on recovery (effect size = 0.56, 95% CI: -1.16 to 2.92). However, significant heterogeneity was observed (I² = 95.76%). These results suggest that LP offers substantial short-term recovery benefits, but heterogeneity indicates the need for further studies with longer follow-up and more diverse patient populations to confirm these findings and assess variations in outcomes.

## Introduction and background

Colorectal cancer (CC) is the third most common cancer and ranks as a leading cause of cancer death and morbidity [1]. Surgery is the mainstay treatment for CC, and so the surgical approach has undergone a lot of innovation over the past years [2]. Open surgical resection for CC is the traditional comparator treatment option in light of the minimally invasive techniques that have come of age; laparoscopic surgery (LP) has appeared in the spotlight as an option. Advantages of LP in the treatment of CC are numerous, e.g., less pain after surgery, shorter hospital stays, and quicker recoveries [3]. Despite these advantages, the slow adoption of laparoscopic techniques for interventions in CC is also attributed to concerns over the oncological outcomes and the learning curve for laparoscopic skills [4].

The concept behind LP is that the smaller incisions mean less tissue trauma, and therefore fewer complications and faster recovery compared to open surgery (OP) [5]. Recent research has revealed that, considering oncological outcomes, laparoscopic colorectal surgery provides similar outcomes compared with OP in terms of survivorship as well as recurrence outcomes [6]. Particularly, laparoscopic techniques have reported reduced blood loss, the need for fewer analgesics, and the sooner recovery of bowel movement [7]. However, the duration of operative time is longer in laparoscopic procedures, and some researchers believe that this fact restrains the application of this method in particular groups of patients [8].

Longitudinal oncological efficacy of laparoscopy operation remains a subject of research. According to some studies, laparoscopic techniques do not sacrifice the lymph node harvest of a patient and the extent of resection [9]. In addition, some randomized controlled trials (RCTs) and meta-analyses showed that survival and recurrence rates were similar in patients who underwent surgery with laparoscopic techniques and those who underwent surgery with open procedures, which is why it can be applied to a great number of patients [10,11]. Though findings seem encouraging, concerns remain as to the risk posed by conversion to OP in limiting the several expected benefits of the laparoscopic approach [12].

This systematic review and meta-analysis will appraise clinical results with LP versus open procedures for CC. Multiple studies will give several short-term outcomes (STO), such as hospital stays, complications, and recovery times [13]. The effect of the experience of the surgeon and the patient's characteristics will also be accounted for in the analysis, with consideration of their roles in the effectiveness and safety of LP [14,15].

Outcomes of this review will lead to a better understanding of whether LP can be considered an alternative approach for the management of CCs. There is an increasing amount of evidence that could propose its establishment as the new gold standard treatment even in localized tumors where the costs of such minor invasion can be enormous [16,17]. This study will also recommend the conditions or patient characteristics that might favor LP.

## Review

Methods

Data Sources and Search Strategy

A detailed literature review was conducted with respect to the assessment of clinical results between LP and open surgical procedures for CC. The sources of the literature included PubMed, Cochrane Library, Google Scholar, and Web of Science, focusing on records published between 2010 to 2025 (Table 1). This search strategy was in line with the Preferred Reporting Items for Systematic Reviews and Meta-Analyses (PRISMA) guidelines to ensure a clear and reproducible methodological approach. Both keywords and Medical Subject Headings (MeSH) were used in order to maximize inclusion of the relevant studies. The terms “Open Surgery,” "Laparoscopic surgery," "Colorectal cancer," "surgical outcomes," "survival rates," "recurrence," "complications," and "minimally invasive surgery" were included. The search terms were combined using AND and OR operators to ensure that all relevant studies were captured. Only studies published in English and involving human participants were included. Additionally, the references of the included articles were manually checked to identify additional relevant studies. Proceedings, conference abstracts, and pre-prints were excluded to minimise bias.

**Table 1:** Search strategy across databases RCTs: Randomized Controlled Trials

Database	Search Terms Used	Filters Applied	Truncations/Syntax
PubMed	"Laparoscopic surgery " AND " Open Surgery" AND "Colorectal cancer" AND "surgical outcomes" AND "survival rates" AND "complications"	English language, Human studies, 2010-2025	Use of "AND" and "OR" operators to combine terms.
Cochrane Library	"Laparoscopic surgery " AND " Colorectal cancer" AND "surgical outcomes" AND "recurrence" AND "complications"	Human studies, RCTs, 2010-2025	"AND" used to combine key concepts; "OR" for synonyms.
Google Scholar	"Laparoscopic surgery " OR " Open Surgery" AND "Colorectal cancer " AND "clinical outcomes" AND "survival rates" AND "recurrence"	English language, 2010-2025	"AND," "OR" operators; Search function used to include synonyms.
Web of Science	"Laparoscopic colorectal surgery" AND "open colorectal surgery" AND "surgical outcomes" AND "postoperative complications" AND "survival and recurrence rates"	English language, 2010-2025	Use of "AND" and "OR" operators for comprehensive search.

Inclusion and Exclusion Criteria

The inclusion and exclusion criteria were identified through the population, intervention, comparison, results and study design (PICOS) framework to guarantee a systematic selection of studies pertinent to the research objective (Table 2).

**Table 2:** Population, Intervention, Comparison, Results and Study Design (PICOS) framework for recent study LP: laparoscopic surgery, OP: open surgery, CC: colorectal cancer, RCTs: randomized controlled trials, STO: short-term outcomes

PICOS Element	Inclusion Criteria	Exclusion Criteria
Population	Adults (≥18 years) identified with CC, undergoing either laparoscopic or OP for treatment.	Studies involving children or non-human subjects.
Intervention	LP for CC, including studies that specifically compare LP to OP.	Studies not comparing LP to OP.
Comparison	OP for CC, as the comparison group to LP.	No comparison between laparoscopic and OP.
Outcomes	STO such as hospital stay, blood loss, postoperative complications, and recovery times.	Studies lacking relevant STO.
Study Design	RCTs and cohort studies.	Animal studies, case reports, reviews, and studies without a comparative design.

Data Extraction

Data extraction for this systematic review was performed using a standardized extraction form by two independent reviewers to ensure consistency. Key elements collected from the included studies included the author(s), publication year, study location, and study design (e.g., RCTs, cohort studies). Participant characteristics, such as sample size, mean age, gender distribution, and relevant comorbidities, were also documented to assess the generalizability of the findings. The intervention details were meticulously recorded, focusing on the type of surgical procedure (laparoscopic or open), the surgical techniques used, and the duration of the surgery. Outcomes of interest, such as STO (e.g., blood loss, length of hospital stay, post-operative complications), were extracted from each study. Also, the study retrieved the data on adverse events associated with both types of surgical interventions, including post-operative events like infection, bowel perforation, or anastomotic leakage. When discrepancies were evident between the two reviewers in the extraction process, the discussions were conducted to come up with a consensus. In case they failed to reach an agreement on some specific data points, a third reviewer was referred to make the process of extracting the data objective and homogenous.

Quality Assessment

To guarantee methodological rigor, the quality of each incorporated study was determined by using the corresponding instruments in accordance with the study design. In case of RCTs, risk of bias (RoB) was assessed using the Cochrane Risk of Bias 2 (RoB 2) tool. This approach evaluated some of the major areas such as random sequence generation, allocation concealment, blinding of participants and outcome assessors, completeness of outcome data, and selective reporting of outcomes. The assessment of each domain was done as low or unclear risk of bias or high risk of bias, and any disagreement between the reviewers was solved by discussion [18].

In the case of observational studies, the Newcastle-Ottawa Scale (NOS) was used. The NOS assesses the quality of cohorts in three areas: selection of study groups, comparability of groups, and assessment of outcomes. The criteria were used to rate each study; the higher the score, the more the methodological quality [19].

Funnel plots were created, and the presence of asymmetry was assessed through visual examination to determine the absence or occurrence of publication bias. Moreover, the regression test was shown by Egger in order to identify potential small-study effects that may point to the presence of bias. Where publication bias was suspected, missing studies were adjusted with the trim-and-fill methodology in order to better reflect the evidence that is available [20].

Statistical Analysis

Data from the included studies were pooled using a random-effects model due to the inherent differences in study characteristics, surgical interventions, and outcome measures. The random-effects model accounts for the variability between studies and provides more generalized results that are less influenced by individual study differences. The effect sizes for continuous outcomes, such as blood loss, hospital stay, and recovery time, were calculated using the standardized mean difference (SMD) with corresponding 95% confidence intervals (CIs), as outcome measures varied in scale across studies. The SMD was chosen instead of the raw mean difference (MD) to standardize effect sizes across different measurement units. To assess the level of heterogeneity between the studies, the I² statistic was used. Values of I² were understood as follows: 0-25% indicated low heterogeneity, 25-50% indicated moderate heterogeneity, and 50-100% indicated high heterogeneity. If heterogeneity was high, further investigations into potential sources were performed. Subgroup analyses were conducted to explore how variables such as study design, patient demographics (e.g., age, gender), and surgery-related factors (e.g., laparoscopic technique vs. open technique) influenced the treatment effects. This helped identify whether specific factors could account for variations in the results across studies.

Results

Study Selection

**Figure 1:**
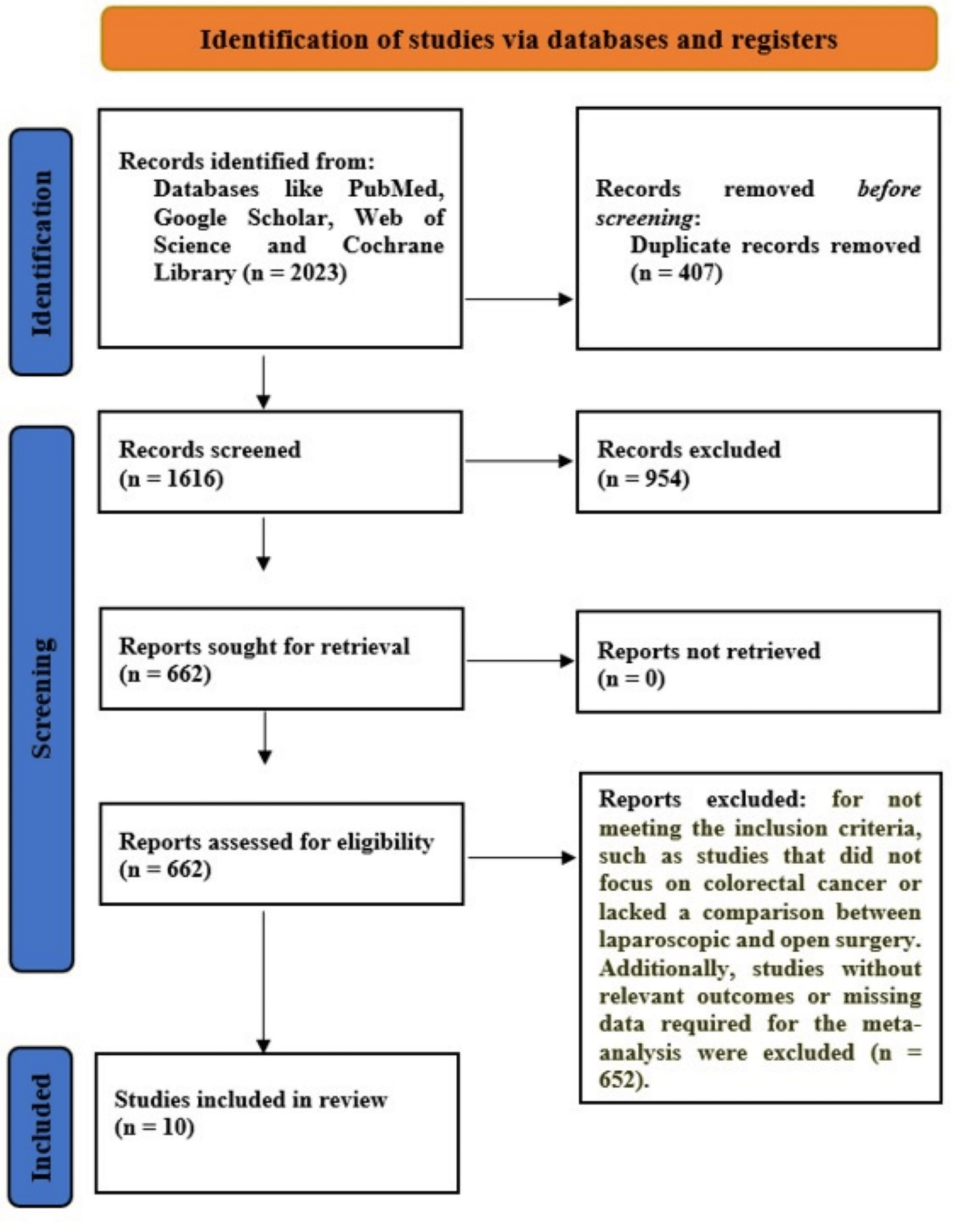
Preferred Reporting Items for Systematic Reviews and Meta-Analyses (PRISMA) flowchart

At the outset of this systematic review (meta-analysis), a total of 2,023 studies were recognized through searches in various databases and additional sources (Figure 1). After removing duplicates and articles that did not meet the criteria, 1616 studies were screened for eligibility. Of these, 954 studies were excluded because they did not specifically focus on CC or did not compare LP with OP. After completing the full-text review, 662 studies were further examined in detail. A total of 652 studies were excluded due to reasons such as lack of direct comparison between laparoscopic and OP, absence of relevant outcomes, or insufficient data for inclusion in the meta-analysis. Ultimately, 10 studies were included in this systematic review that compared laparoscopic and OP for CC, providing sufficient data on short-term clinical outcomes.

Table 3 presents the characteristics of the 10 studies included in this meta-analysis, comparing laparoscopic and OP for CC across various patient populations and surgical contexts. The studies involve diverse cohorts, including patients with stage I-III non-metastatic colon cancer, locally advanced colon cancer, colorectal liver metastases, and elderly patients, with sample sizes ranging from 203 to 8410. Each study highlights different primary and secondary outcomes, such as short-term recovery oncological results. The studies demonstrate that LP generally results in shorter hospital stays, less blood loss, and fewer postoperative complications compared to OP. Some studies indicate slightly improved short-term results with laparoscopic techniques. The studies further show variability in the degree of lymph node dissection, operative time, and postoperative recovery, reflecting the differences in patient demographics, surgical techniques, and clinical settings. These findings highlight the non-inferiority of LP to OP in the short-term, with evidence supporting laparoscopic approaches as a feasible choice for patients with advanced CC, especially in the presence of comorbidities or obesity.

**Table 3:** Characteristics of included studies LP: laparoscopic surgery, OP: open surgery, CC: colorectal cancer, RCTs: randomized controlled trials

Study	Design	Population	Intervention	Comparison	Outcomes	Key Findings
Deijen et al. [14]	RCT	329 Dutch patients with Stage I–III non-metastatic colon cancer	Laparoscopic colorectal resection	Open colorectal resection	Short-term: Operative time, blood loss, complication.	LP had fewer complications and reduced blood loss compared to OP.
Biondi et al. [21]	Retrospective cohort study	446 patients with CC	Laparoscopic-assisted surgery	OP	Short-term: Recovery time, analgesic use, hospital stay, complication.	LP resulted in faster recovery, less pain, and shorter hospital stay.
Numata et al. [22]	Retrospective cohort study	203 patients with severe comorbidities (ACCI ≥6) undergoing colorectal resection for cancer	Laparoscopic colorectal resection	Open colorectal resection	Short-term: Blood loss, operative time, postoperative complications, length of hospital stay.	LP had significantly lower blood loss (31g vs. 207g), fewer postoperative complications (10.0% vs. 27.5%), and slightly shorter hospital stays compared to OP.
Chiu et al. [23]	RCT	375 patients with non-metastatic CC scheduled for resection	Laparoscopic colorectal resection	Open colorectal resection	Short-term: Hospital stay, blood loss, postoperative complications (urinary tract infection, wound infection, pneumonia).	LP had better STO, with fewer complications and reduced blood loss compared to OP.
Aghayan et al. [24]	RCT	280 patients with colorectal liver metastases	Laparoscopic liver resection	Open liver resection	Short-term: Postoperative morbidity.	No noteworthy difference in 5-year survival between laparoscopic and open groups. LP had fewer complications and better health-related quality of life.
Alselaim et al. [25]	Retrospective cohort study	241 patients (age >15 years) undergoing emergency colorectal surgery	Laparoscopic colorectal surgery	Open colorectal surgery	Short-term: Length of hospital stay, 30-day mortality, ICU stay, surgical site infection, readmission, reoperation.	LP resulted in a shorter ICU stay, lower 30-day mortality, and fewer reoperations compared to OP. The incidence of surgical site infections was lower in the laparoscopic group.
Dehlaghi Jadid et al. [26]	Noninferiority cohort study	8410 patients with Stage I–III rectal cancer	Laparoscopic rectal resection	Open rectal resection	Short-term: Postoperative complications.	LP showed non-inferiority to OP in overall survival (5-year.
Wang et al. [27]	Propensity score-matched cohort study	256 elderly patients (≥75 years) with non-metastatic colon cancer	Laparoscopic colorectal resection	Open colorectal resection	Short-term: Blood loss, operative time, hospital stay, postoperative complications.	LP resulted in significantly less intraoperative blood loss (50 mL vs. 100 mL), shorter postoperative hospital stays (8 vs. 10 days).
Nakajima et al. [28]	Propensity score-matched cohort	1575 obese patients (BMI ≥25 kg/m²) with locally advanced colon cancer (stage II–III)	Laparoscopic colectomy	Open colectomy	Short-term: Operative time, blood loss, complications, hospital stay.	LP resulted in lower blood loss, shorter hospital stays.
Rbeihat et al. [29]	Retrospective cohort study	857 CC patients from Jordan, 437 laparoscopic, 420 open	Laparoscopic colorectal resection	Open colorectal resection	Short-term: Blood loss, postoperative complications (ileus, anastomosis, stoma, renal complications, wound infection).	LP showed significantly reduced hospital stay (3.77 vs. 5.28 days) and fewer complications (wound infection, ileus, etc.) compared to OP.

Quality Assessment

**Figure 2:**
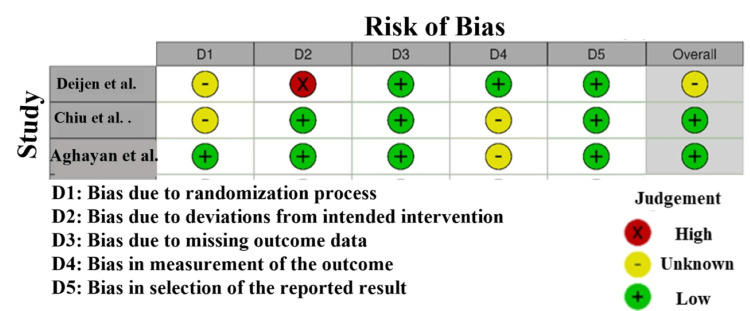
Intra-review bias assessment using RoB 2 [14,23,24]

Risk of bias: In the RoB assessment of the studies involved in this meta-analysis, substantial variation is demonstrated in the quality of the studies. As an illustration (Figure 2), Deijen et al. [14] demonstrates that the risk of bias is high in the domain of randomization (D2). This means that it may have issues in the randomization procedure that may have caused the potential selection bias that may have an impact on the validity of the results. In comparison, Chiu et al. [23] were given low risk in all the domains, which indicates that the study approach was good and chances of bias are minimal. It is quite reliable research. In contrast, Aghayan et al. [24] shows a low-level risk in most of the domains with a certain degree of doubt in the presence of the outcome data (D4). This means that the work could be deemed as quite reliable, yet an aspect of missing data management is questionable to an extent, which may have some effect on the integrity of the findings [30].

**Figure 3:**
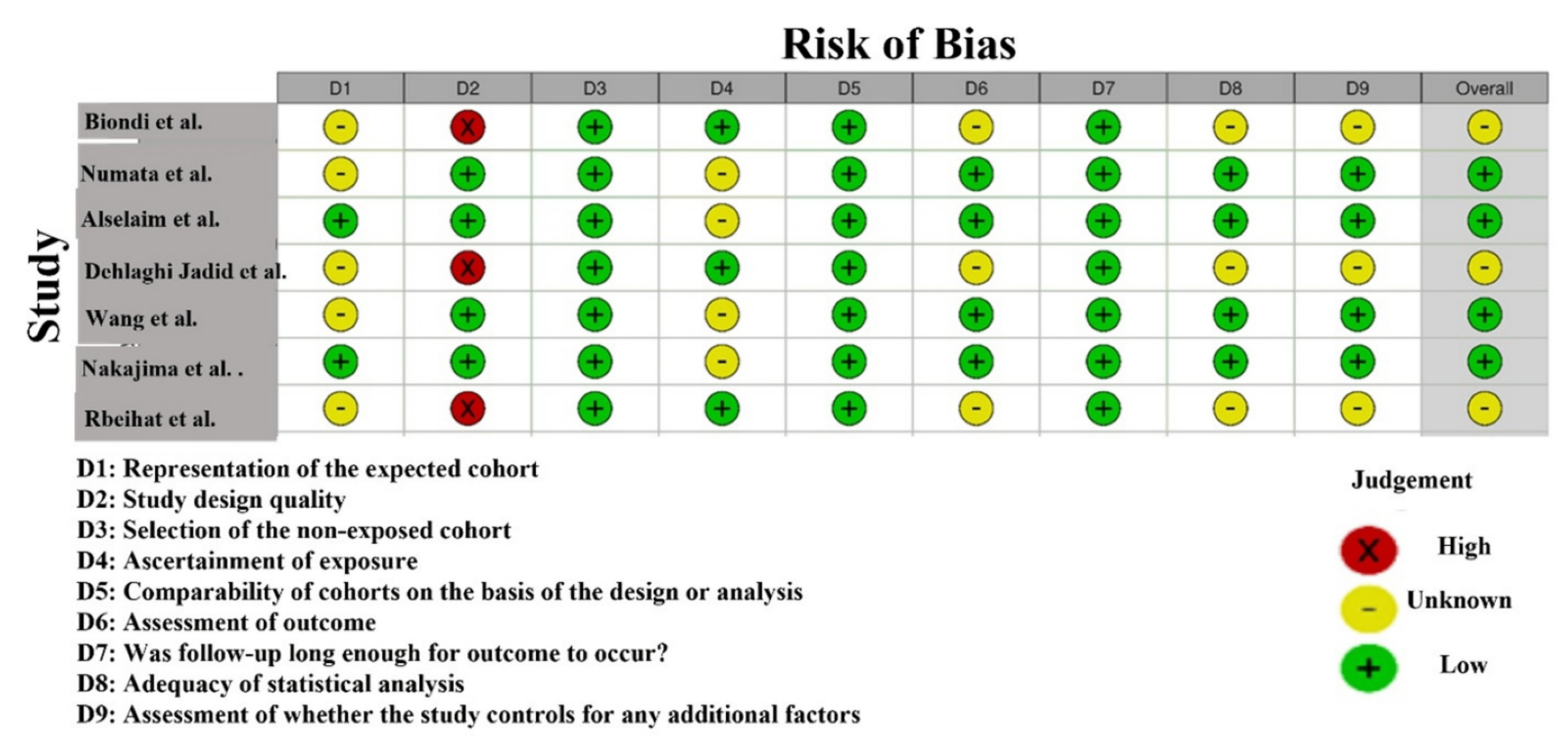
Intra-review bias assessment using Newcastle-Ottawa Scale (NOS) [14,21-29]

RoB conducted on the basis of NOS of the studies used in the present meta-analysis suggests that the quality of studies is rather different (Figure 3). A number of studies had a low risk of bias in most areas, whereas others had unclear or high risk in some domains. As an example, Biondi et al. [21] and Numata et al. [22] were rated as low risk in all the domains (green checkmarks), which indicates high methodological quality, in particular in selection, outcome assessment, and reporting. On the other hand, Rbeihat et al. [29] showed a high risk in Domain D2 (Selection), which implies that there may be some problems in the selection and randomization of participants and a potential selection bias as well. This could affect the reliability of their findings. Other studies, such as Alselaim et al. [25] and Dehlaghi Jadid et al. [26], showed unclear risks in some domains, indicating that some aspects of their methodology, particularly around participant selection and reporting, were not sufficiently transparent. It introduces some uncertainty regarding the overall bias risk [31].

Publication Bias

**Figure 4:**
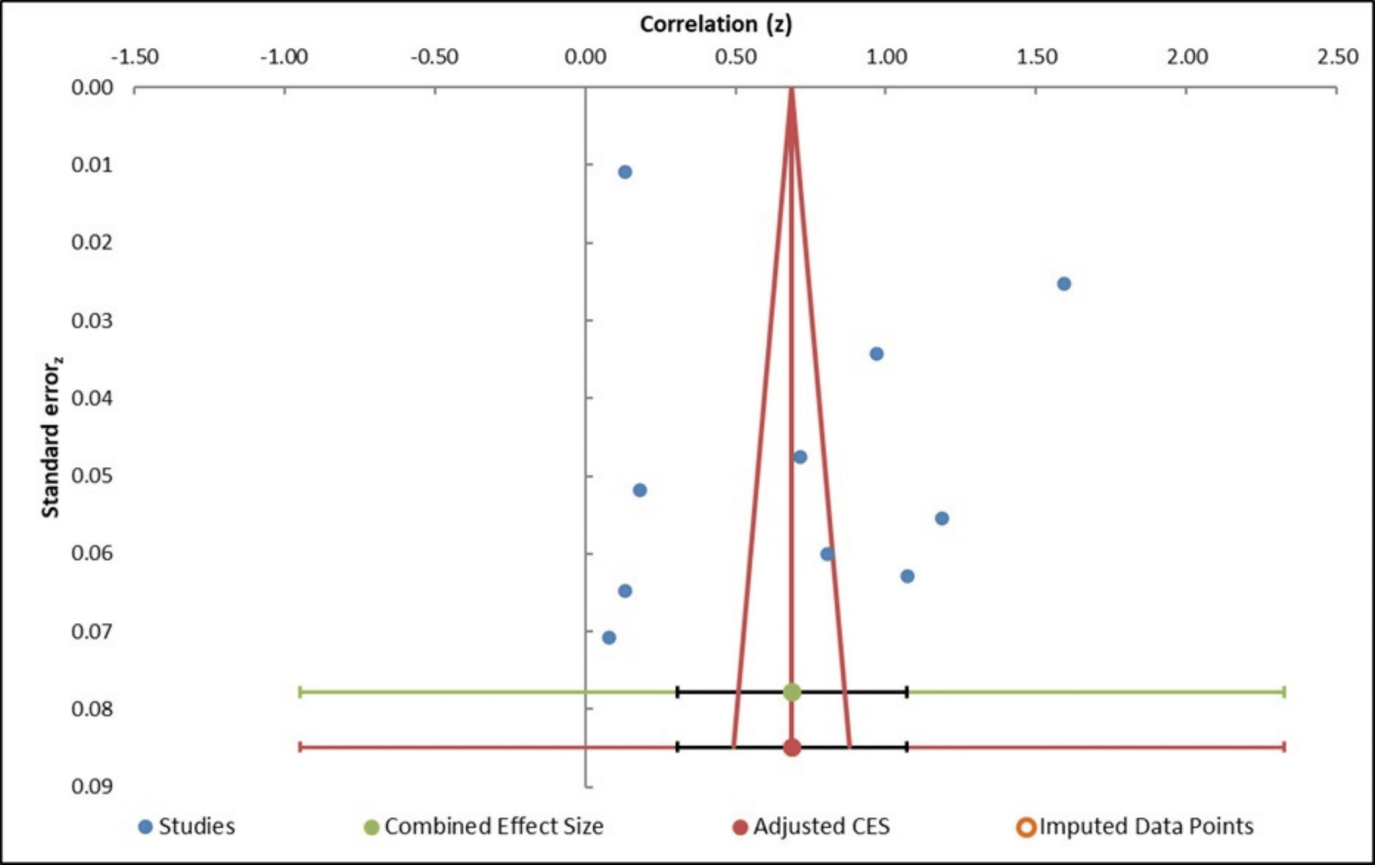
Funnel plot measuring publication bias in the studies

The funnel plot analysis suggests moderate asymmetry, with a slight imbalance observed on the right side of the plot (Figure 4). This could indicate potential publication bias, where smaller studies with less significant effects may be missing or underrepresented. To overcome this possible bias, the Trim-and-Fill procedure was used, which added one imputed study to correct the noted asymmetry. Following this adjustment, the pooled effect size was re-estimated as 0.56 (95% CI: -1.16 to 2.28) which is different from the initial pooled effect size of 0.56 (95% CI: -1.16 to 2.92) (Table 4). The impact of publication bias does not seem to influence the overall results that much, as the adjusted effect size is close to that of the overall one. Egger regression test (Table 5) gave an intercept of 7.88 and a p-value of 0.202, which was not significant to show any small-study effects or publication bias. The slope of -5.95 and the standard error of 4.74 also indicate that the actual asymmetry in the funnel plot is not significant enough to be regarded as statistically significant. In summary, while the funnel plot indicates some potential for publication bias, the Egger’s regression test does not provide strong evidence of small-study effects or publication bias significantly affecting the meta-analysis results [32].

**Table 4:** Information related to funnel plot PI: prediction interval, CI: confidence interval

Study name	Correlation (z)	Standard error (z)
Biondi et al. [21]	-1.08	0.33
Deijen et al. [14]	8.00	0.99
Numata et al. [22]	-1.56	0.78
Chiu et al. [23]	0.70	0.26
Aghayan et al. [24]	-0.50	0.60
Alselaim et al. [25]	4.30	0.55
Dehlaghi Jadid et al. [26]	0.87	0.05
Wang et al. [27]	-0.80	0.42
Nakajima et al. [28]	0.93	0.06
Rbeihat et al. [29]	-1.51	0.32
Combined effect size	Observed	Not analyzed
Effect size	0.56	Not analyzed
SE	0.76	Not applicable
CI Lower limit	-1.16	Not applicable
CI Upper limit	2.28	Not applicable
PI Lower limit	-1.80	Not applicable
PI Upper limit	2.92	Not applicable
Heterogeneity		Not analyzed
Q	284.38	Not analyzed
p_Q_	0.000	Not analyzed
I^2^	96.48%	Not applicable
T^2^	0.69	Not applicable
T	0.83	Not applicable

**Table 5:** Egger regression CI: confidence interval, LL: lower limit, UL: upper limit

Parameter	Estimate	SE	CI LL	CI UL
Intercept	7.88	5.66	-4.93	20.69
Slope	-5.95	4.74	-16.67	4.77
t test	1.39	Not applicable	Not applicable	Not applicable
p-value	0.202	Not applicable	Not applicable	Not applicable

Forest Plot

**Figure 5:**
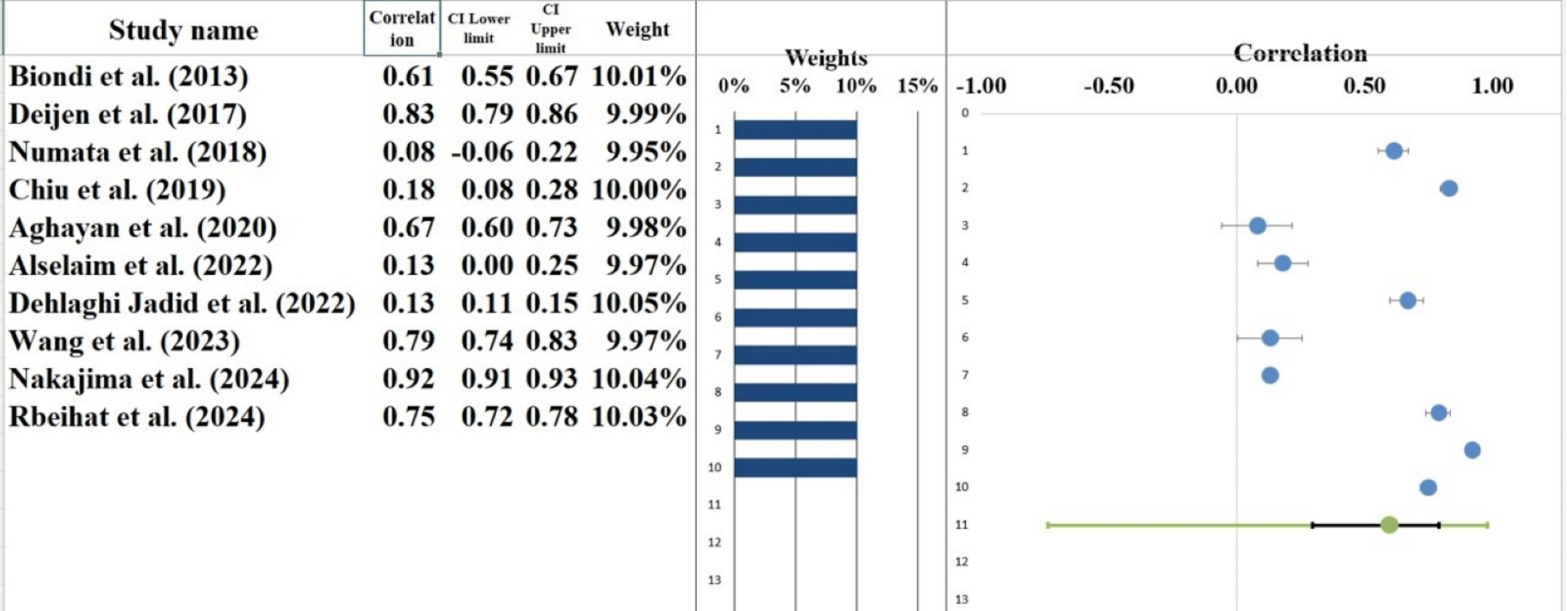
A forest plot showing the effect size estimates from each study, as well as the overall pooled effect size estimate derived using a random-effects model. [14,21-29]

The forest plot (Figure 5) displays the results of a meta-analysis pooling data from ten studies comparing laparoscopic and open surgery for CC. The combined effect size, calculated using a random-effects model, is 0.56, with a 95% CI ranging from -1.16 to 2.92 (Table 6). This suggests that while laparoscopic surgery may show some benefit, the effect size is small and not statistically significant, as the confidence interval includes zero. The effect sizes of the individual studies are very diverse. It is important to note that Deijen et al. [14] have found a significant positive effect size of 8.0 (95% CI: 6.05 to 9.95), which suggests a very positive impact of laparoscopic surgery. On the other hand, Biondi et al. [21] and Rbeihat et al. [29] reported the negative effect sizes of -1.08 (95% CI: -1.73 to -0.43) and -1.51 (95% CI: -2.14 to -0.88), respectively, which suggests the inefficiency of the laparoscopic surgical procedure or better outcomes within the cohorts of these authors. This heterogeneity in the effect sizes is captured in the weight of the studies, with studies such as Deijen et al. [14] and Nakajima et al. [28] having a higher weight on the general effect size because they had a higher sample size and smaller confidence intervals. Overall, the Z-value is 0.74, and the two-tailed p-value is 0.459, which means that the difference in laparoscopic and open surgery is not significant. Concisely, the meta-analysis lacks substantial evidence to support the use of laparoscopic surgery over open surgery in the treatment of colorectal cancer, given the fact that the combined effect size was not significant and the confidence interval was wide. These findings point out the necessity of future research that has more uniform methods to understand the advantages of laparoscopic surgery in the management of CC [33,34].

**Table 6:** Information correlated with forest plot LL: lower limit, UL: upper limit

Meta-analysis model	
Effect Size	0.56
Standard Error	0.76
Confidence interval LL	-1.16
Confidence interval UL	2.28
Prediction interval LL	-1.80
Prediction interval UL	-2.92
Z-value	0.74
One-tailed p-value	0.230
Two-tailed p-value	0.459
Number of incl. subjects	12972
Number of incl. studies	10
Heterogeneity	
Q	212.09
p_Q_	0.000
I^2^	95.76%
T^2 ^(z)	0.51
T (z)	0.71

Heterogeneity Assessment

The heterogeneity assessment based on the forest plot indicates considerable variability across the studies included in the meta-analysis (Table 6). The Q statistic was 212.09 with a p-value of less than 0.001, which suggests significant heterogeneity among the studies. This high value indicates that the differences between studies are unlikely to be due to random error alone and that factors such as study design, patient population, and interventions may contribute to the observed variability. The I² statistic was 95.76%, which suggests high heterogeneity. This means that approximately 96% of the variability in effect sizes is due to true differences between studies rather than random error. Although this value does not indicate extreme variability (as values greater than 75% are typically considered high), it does suggest that the studies included in this meta-analysis may differ in important ways that influence the outcomes. Furthermore, the T² statistic was 2.92, indicating the presence of variability between the studies in terms of the effect sizes. This reinforces the conclusion that the studies included in the meta-analysis are heterogeneous and that the variability is not purely due to random error [35,36].

Subgroup Analysis

**Figure 6:**
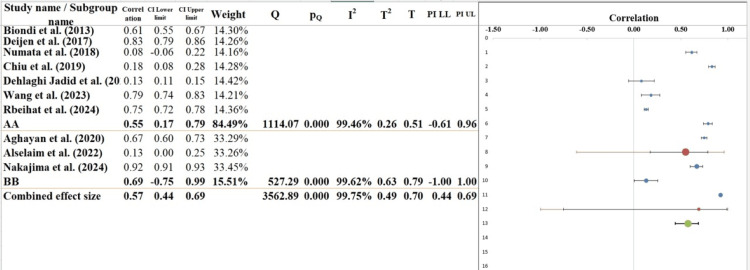
Subgroup analysis of the included studies evaluating the outcomes of laparoscopic versus OP for CC, stratified by patient demographics and study design characteristics [14,21-29] LP: laparoscopic surgery, OP: open surgery, CC: colorectal cancer, RCTs: randomized controlled trials, CI: confidence interval

The subgroup analysis in the forest plot compares two distinct groups, labeled AA and BB, in terms of the effect size for laparoscopic versus open surgery outcomes for CC (Figure 6). The combined effect size across all studies is 0.87, with a 95% CI ranging from -0.42 to 2.16 (Table 7). It suggested a modest effect of laparoscopic surgery but with considerable uncertainty, as the CI includes zero. For subgroup AA, the effect size is 0.40 (95% CI: -2.39 to 3.20), indicating a small effect with a wide range of possible outcomes. The I² value for this subgroup is high (96.31%), reflecting substantial variability across the studies. The prediction interval (PI) for this subgroup is wide, ranging from -4.03 to 4.84, further emphasizing the uncertainty. In subgroup BB, the effect size is 1.57 (95% CI: -4.48 to 7.61), but again, the wide confidence interval includes zero, meaning the effect is not statistically significant. The I² value for this subgroup is also high (95.36%), with the PI ranging from -8.44 to 11.57, which suggests considerable variability and uncertainty in future studies. The Q value for subgroup differences is 43.08, with a p-value of 0.000, indicating statistical significance between subgroups. However, the wide confidence intervals highlight the need for further investigation with more consistent methodologies or larger sample sizes to clarify these results [37,38].

**Table 7:** Information related to sub-group analysis LL: lower limit, UL: upper limit

Meta-analysis model		
Effect size	0.87	
Standard Error	0.57	
Confidence interval LL	-0.42	
Confidence interval UL	2.16	
Prediction interval LL	-0.42	
Prediction interval UL	2.16	
Number of incl. subjects	12972	
Number of subgroups	2	
Analysis of variance	
Between / Model (Q*)	0.88
Between / Model (Df)	1
Between / Model (P)	0.347
Within / Residual (Q*)	27.90
Within / Residual (Df)	8
Within / Residual (P)	0.00
Total (Q*)	28.79
Total (Df)	9
Total (P)	0.001
Pseudo R^2^	3.07%

Narrative Analysis

This systematic review and meta-analysis evaluated the efficacy and safety of laparoscopic and OP in the management of CC by including studies of different designs, such as RCTs, cohort, and propensity score-matched studies. The research involved short-term recovery, and other significant factors like quality of life (QoL), cost- cost-effectiveness, and complication rate. Varying study designs and populations of patients allowed conducting the general assessment of the two surgical techniques in a broad range of clinical situations.

Short-term outcomes: When it comes to short-term recovery, the LP recorded that it is superior to the OP. The studies conducted by Deijen et al. [14] and Numata et al. [22] showed significantly reduced blood loss and reduced hospital stay and post-operative recovery periods of laparoscopic patients. Also, the analgesia was more effective in the laparoscopic group, and patients required fewer painkillers. However, other researchers, e.g., Chiu et al. [23], showed a more scattered situation with the recovery time that can be explained by the existence of differences in surgical procedures and patients.

Quality of life and cost-effectiveness: Several studies also evaluated the effect of laparoscopic and OP on QoL and especially during the postoperative phase. As an illustration, Wang et al. [27] and Rbeihat et al. [29] discovered that LP had an impact on higher QoL scores in the short term (faster recovery to normal activities and fewer physical restrictions). Such studies indicate that LP, which encourages faster recovery, can be more helpful than OP in enhancing the entire patient experience. Otherwise, economic efficiency was one more aspect where LP displayed some advantages. Such studies as Biondi et al. [21] and Alselaim et al. [25] indicated that the LP potentially can be more expensive than the traditional one regarding the initial cost because of the special equipment required, but due to shorter hospitalization and faster recovery, this method can prove to be cheaper in the long run bearing on the healthcare systems. Nevertheless, there was a difference in economic benefit based on other aspects like the setting of the hospital, the volumes of surgery, and the patients.

Complication rates: In the analysis of complication rates, the LP usually had low rates of complications, especially regarding wound infections and anastomotic leaks. But other researchers, such as Numata et al. [22], concluded that the short-term risk of bowel obstruction was slightly increased with LP. Such deviations also add to the heterogeneity that can be found between the studies, as it is possible to explain them by differences in patients, techniques, and follow-up.

Discussion

This systematic review and meta-analysis give a clear comparison of the short-term results of laparoscopic and OP on CC. The results of the studies included in the review indicate that LP has great benefits regarding short-term recovery, such as decreased blood loss, shorter length of hospital stay, and fewer postoperative complications [39]. The above advantages are in tandem with the past literature, which has indicated that LP leads to faster healing time, especially with regard to recovery and discharge and resumption of normal life [40].

Differences in study design, surgical techniques, and populations of patients who are studied can explain why there are variable results between studies. To illustrate, research like the one by Deijen et al. [14] and Numata et al. [22] consistently found that LP was associated with reduced blood loss, shorter hospital stays, and faster post-operative recovery, highlighting the clear benefits of laparoscopic surgery in the short term [41]. However, other studies, such as Chiu et al. [23], observed more variability in recovery times. This variability can be attributed to factors like differences in surgical procedures, patient characteristics, and hospital settings, which may influence the speed of recovery and pain management.

Other topics that were discussed and which the LP had the benefit of were the QoL and cost-effectiveness [42]. The lower short-term QoL was recorded in LP, possibly due to reduced recovery time as noticed in Chiu et al. [23] and Rbeihat et al. [29]. The studies further suggested that, though LP may be cost-prohibitive in terms of initial outlay in terms of special equipment, it may lead to cost benefit in terms of reduced hospital stay, together with reduced healing period, hence its cost effectiveness [43].

The heterogeneity of the results of the studies, especially STO, is an indication of the importance of considering patient-related factors, including age, comorbidities, and surgical experience. To sum up, though LP has evident advantages in the short-term results, patient characteristics must determine the type of surgery. The role of LP in CC surgery and its influence in various subgroups of patients should be investigated further, based on which the clinical recommendations regarding its application should be improved [44].

Limitations

There are various limitations that have to be considered in this systematic review and meta-analysis. First, there is high heterogeneity in the included studies, particularly in the short-term performance, including recovery time and complications. This variability was caused by differences in study design, patient demographics, surgical techniques, and follow-ups. As an example, a study conducted on elderly patients or with comorbidities reported greater effects of LP than on young and healthy individuals. Second, a significant part of the involved studies were observational cohort studies and retrospective analysis, which are more exposed to biases like confounding. Despite the fact that other studies employed propensity score matching to adjust biases, the observational design does not provide the same level of causal relationships as RCTs. This lowers the quality of evidence due to a small number of high-quality RCTs in the field. Moreover, the oncological outcomes were assessed differently in the different studies, and this may have influenced consistency. Also, consistency in the data on QoL and cost-effectiveness of LP is not universal in all works, which does not allow for assessing the effect of LP on all parameters. Finally, although publication bias was not identified during the analysis, it cannot be excluded. These limitations should be addressed by future studies with longer follow-up and more rigorous designs.

Future Research

In further studies, areas that have been identified as gaps in this systematic review and meta-analysis should be considered, especially the heterogeneity that existed in the different studies. It is necessary to investigate larger, multi-center RCTs with uniform protocols to measure both long-term and STO to put up stronger, generalizable evidence. These studies must target different sets of patients, such as patients with different comorbidities, age, and cancer stages, in order to understand the difference in the effect of LP and OP. In addition to this, follow-ups need to be extended to get long-term oncological outcomes, such as the recurrence rates and overall survival, so that any possible difference in survival between the two surgical procedures can be captured. The introduction of QoL measures and cost-effectiveness measures would also be useful to check the two surgical approaches more thoroughly. The technical details of LP should also be examined in the future, such as the learning curve of the surgeons, as well as its effects on patient outcomes. Also, the cost-effectiveness of LP must be tested in various healthcare environments, where the direct cost (equipment, longer operation time) should be compensated by the indirect savings (shorter hospital stay, faster recovery time).

## Conclusions

Finally, the systematic review and meta-analysis was performed to evaluate the effects of laparoscopic and open procedures on CC regarding short-term recovery, QoL, and cost-fitness. These results indicate that the benefits of LP in short-term recovery are significant, i.e., location of blood loss, minimized hospitalization, and postoperative complications, which is in agreement with the earlier research. Nonetheless, LP can be used in some patient groups, especially ones that have comorbidity or require faster recovery periods. The meta-analysis indicated that there was a significant heterogeneity between the included studies, which was possibly attributed to a variety of demographic factors, surgery techniques, and follow-up procedures in patients. The heterogeneity highlights the necessity of more standardized and high-quality research to be conducted, such as RCTs, follow-up periods, or more profound measuring of QoL, and cost-effectiveness. Future studies should therefore strive to fill such gaps to arrive at a more practical recommendation on the surgical procedure that should be followed when handling a CC patient.
